# The ancient Virus World and evolution of cells

**DOI:** 10.1186/1745-6150-1-29

**Published:** 2006-09-19

**Authors:** Eugene V Koonin, Tatiana G Senkevich, Valerian V Dolja

**Affiliations:** 1 National Center for Biotechnology Information, National Library of Medicine, USA; 2 Laboratory of Viral Diseases, National Institute of Allergy and Infectious Diseases, National Institutes of Health, Bethesda, MD 20894, USA; 3 Department of Botany and Plant Pathology and Center for Genome Research and Biocomputing, Oregon State University, Corvallis, OR 97331, USA

## Abstract

**Background:**

Recent advances in genomics of viruses and cellular life forms have greatly stimulated interest in the origins and evolution of viruses and, for the first time, offer an opportunity for a data-driven exploration of the deepest roots of viruses. Here we briefly review the current views of virus evolution and propose a new, coherent scenario that appears to be best compatible with comparative-genomic data and is naturally linked to models of cellular evolution that, from independent considerations, seem to be the most parsimonious among the existing ones.

**Results:**

Several genes coding for key proteins involved in viral replication and morphogenesis as well as the major capsid protein of icosahedral virions are shared by many groups of RNA and DNA viruses but are missing in cellular life forms. On the basis of this key observation and the data on extensive genetic exchange between diverse viruses, we propose the concept of the ancient virus world. The virus world is construed as a distinct contingent of viral genes that continuously retained its identity throughout the entire history of life. Under this concept, the principal lineages of viruses and related selfish agents emerged from the primordial pool of primitive genetic elements, the ancestors of both cellular and viral genes. Thus, notwithstanding the numerous gene exchanges and acquisitions attributed to later stages of evolution, most, if not all, modern viruses and other selfish agents are inferred to descend from elements that belonged to the primordial genetic pool. In this pool, RNA viruses would evolve first, followed by retroid elements, and DNA viruses. The Virus World concept is predicated on a model of early evolution whereby emergence of substantial genetic diversity antedates the advent of full-fledged cells, allowing for extensive gene mixing at this early stage of evolution. We outline a scenario of the origin of the main classes of viruses in conjunction with a specific model of precellular evolution under which the primordial gene pool dwelled in a network of inorganic compartments. Somewhat paradoxically, under this scenario, we surmise that selfish genetic elements ancestral to viruses evolved prior to typical cells, to become intracellular parasites once bacteria and archaea arrived at the scene. Selection against excessively aggressive parasites that would kill off the host ensembles of genetic elements would lead to early evolution of temperate virus-like agents and primitive defense mechanisms, possibly, based on the RNA interference principle. The emergence of the eukaryotic cell is construed as the second melting pot of virus evolution from which the major groups of eukaryotic viruses originated as a result of extensive recombination of genes from various bacteriophages, archaeal viruses, plasmids, and the evolving eukaryotic genomes. Again, this vision is predicated on a specific model of the emergence of eukaryotic cell under which archaeo-bacterial symbiosis was the starting point of eukaryogenesis, a scenario that appears to be best compatible with the data.

**Conclusion:**

The existence of several genes that are central to virus replication and structure, are shared by a broad variety of viruses but are missing from cellular genomes (virus hallmark genes) suggests the model of an ancient virus world, a flow of virus-specific genes that went uninterrupted from the precellular stage of life's evolution to this day. This concept is tightly linked to two key conjectures on evolution of cells: existence of a complex, precellular, compartmentalized but extensively mixing and recombining pool of genes, and origin of the eukaryotic cell by archaeo-bacterial fusion. The virus world concept and these models of major transitions in the evolution of cells provide complementary pieces of an emerging coherent picture of life's history.

**Reviewers:**

W. Ford Doolittle, J. Peter Gogarten, and Arcady Mushegian.

## Open peer review

This article was reviewed by W. Ford Doolittle, J. Peter Gogarten, and Arcady Mushegian.

For the full reviews, please go to the Reviewers' comments section.

## Background

### The extraordinary diversity of viruses

Viruses are ubiquitous companions of cellular life forms: it appears that every cellular organism studied has its own viruses or, at least, virus-like selfish genetic elements [1]. Recent environmental studies have shown that viruses, primarily, bacteriophages, are "most abundant biological entities on the planet" [2], with the total number of virus particles exceeding the number of cells by at least an order of magnitude [3,4]. Viruses actively move between biomes and are thought to be major agents of evolution by virtue of their capacity to operate as vehicles of horizontal gene transfer (HGT) [5].

**Figure 1:**
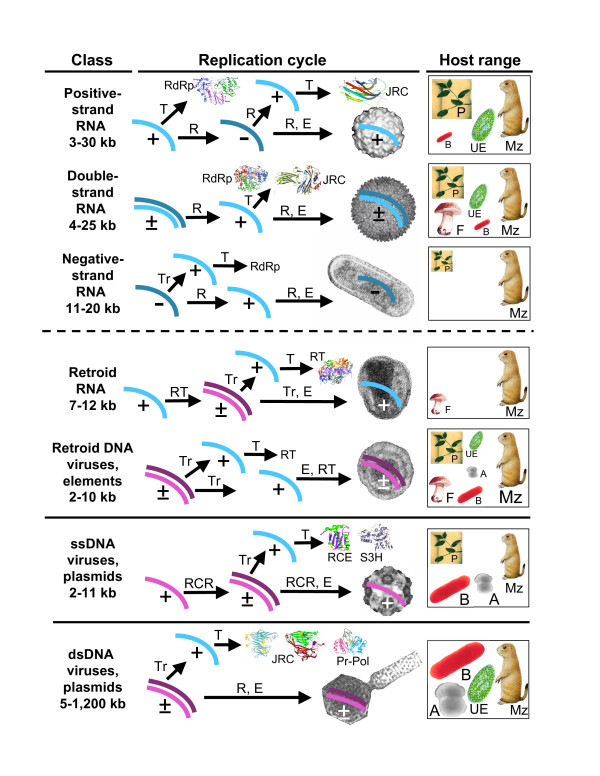
**Viruses and other selfish elements: the replication strategies, genome size distribution, global ecology, and hallmark proteins**. For each class of viruses and related elements, the approximate range of genome sizes is indicated (kb, kilobases). '+' denotes positive strand (same polarity as mRNA) and '-' denotes negative strand. Tr, transcription; T, translation; R, replication; E, encapsidation; A, archaea; B, bacteria; F, fungi; Mz, Metazoa; P, plants; UE, unicellular eukaryotes. For each class of viruses (elements), characteristic structures of hallmark proteins and characteristic electron-microscopic images of viruses are shown. RdRp, RNA-dependent RNA polymerase; JRC, jelly-roll capsid protein; RT, reverse transcriptase; RCRE, rolling-circle replication (initiating) endonuclease. The rightmost panel shows the host range, with the size of the respective image and acronym roughly proportionate to the abundance of the given virus class in the respective taxon.

A remarkable feature of viruses is the diversity of their genetic cycles, in a sharp contrast to the uniformity of the cellular genetic cycle [6-9] (Fig. 1). Viruses with different genome strategies span a vast range of genome sizes (the genomes of the largest known virus, the mimivirus, and the smallest viruses, e.g., circoviruses, differ by three orders of magnitude) and show a non-uniform and non-trivial distribution among the host taxa (Fig. 1). For example, the extraordinary diversity of double-stranded (ds) DNA bacteriophages is in a stark contrast to the absence of bona fide dsDNA viruses in plants. Conversely, RNA viruses are extremely abundant and diverse in plants and animals but are currently represented by only two compact families in bacteria, and so far have not been detected in archaea (Fig. 1).

Given the variety of genetic strategies, genome complexity, and global ecology of viruses, the problem of virus evolution inevitably digresses into a web of interlocking questions. What qualifies as a virus? Are viruses as a whole monophyletic, i.e., ultimately descend from a single primordial virus or polyphyletic, i.e., have multiple origins? If viruses are polyphyletic, how many independent lineages are there? What is the age distribution of different groups of viruses – are they ancient or have they been emerging continuously throughout life's evolution? And, perhaps, the most fundamental questions of all: what is the ultimate origin of viruses and what are the relationships between evolution of viruses and cellular life forms? The recent advances of comparative genomics create the unprecedented opportunity to start tackling these issues by inferring some of the likely answers from sequence and structure data analysis. Here, we address several of these questions but, primarily, the last two, most general ones, in an attempt to outline a coherent scenario of virus origin and evolution and delineate the connections between the evolution of viruses and cellular life forms.

## Results and discussion

### Polyphyly versus monophyly in virus evolution

Comparative genomics provides no evidence of a monophyletic origin of all viruses. Many virus groups simply share no common genes, effectively, ruling out any conventional notion of common origin. When applied to viruses, the notion of "common genes" is not a simple one because commonality is not necessarily limited to clear-cut orthologous relationships between genes that translate into highly significant sequence similarity. Instead, as discussed in the next sections, distant homologous relationships among viral proteins and between viral proteins and their homologs from cellular life forms could convey more complex but important messages on evolution of viruses. This complexity notwithstanding, cases of major virus groups abound that either share no homologous genes under any definition or have in common only distantly related domains with obviously distinct evolutionary trajectories. For example, most of the viruses of hyperthermophilic crenarchaea have literally no genes in common with any other viruses [10,11], whereas RNA viruses share with DNA viruses and plasmids that replicate via the rolling circle mechanism only extremely distant domains in their respective replication proteins.

By contrast, monophyly of several large classes of viruses, including vast assemblages of RNA viruses and complex DNA viruses, can be demonstrated with confidence (Table 1). Some of these monophyletic classes of viruses cross the boundaries set by genome strategies: thus, the retroid class includes both RNA viruses and viruses, mobile elements, and plasmids with DNA genomes, and the rolling circle replication (RCR) class combines ssDNA and dsDNA viruses and plasmids. Furthermore, based on similarities in the structure of RNA replication complexes, along with the presence of homologous, even if distant, replication enzymes, it has been suggested that positive-strand RNA viruses, double-stranded RNA viruses, and retroid elements all have a common origin [9]. On the whole, however, the conclusion seems inevitable that viruses comprise several distinct lines of descent (Table 1).

**Table 1:** The major monophyletic classes of viruses and selfish genetic elements

**Class of viruses**	**Constituent virus lineages**	**Hosts**	**Support for monophyly**	**Refs**
Positive-strand RNA viruses	Superfamily I: picorna-like; superfamily II: alpha-like; superfamily III: flavi-like; the exact affinity of RNA bacteriophages within this class of viruses remains uncertain (possibly, a fourth lineage)	Animals, plants, protists, bacteria (one family of bacteriophages)	Conserved RdRp; JRC in most superfamily 1 viruses, and subsets of superfamilies 2 and 3 viruses. Reconstructed ancestor with RdRp and JRC	[87]
Retroid viruses and elements	Retroviruses, hepadnaviruses, caulimoviruses, badnaviruses; LTR- and nonLTR retroelements; retrons; group II self-splicing introns – the progenitors of eukaryotic spliceosomal introns	Animals, fungi, plants, protists, bacteria, archaea	Conserved RT	[103, 104]
Small DNA viruses, plasmids, and transposons with rolling circle replication	Gemini-, circo-, parvo-, papovaviruses, phages (e.g., φX174), archaeal and bacterial plasmids, eukaryotic helitron transposons	Animals, plants, archaea, bacteria	Conserved RCRE, JRC, S3H (in eukaryotic viruses)	[17, 18, 20]
Tailed bacteriophages (Caudovirales)	Families: Myoviridae (e.g., T4), Podoviridae (e.g., T7), Siphoviridae (e.g., λ)	Bacteria, euryarchaea	Complex, overlapping arrays of genes conserved in subsets of tailed phages; genes of all tailed phages thought to comprise a single pool	[11, 93, 94, 105, 106]
Nucleo-cytoplasmic large DNA viruses (NCLDV)	Poxviruses, asfarviruses, iridoviruses, phycodnaviruses, mimiviruses	Animals, algae, protests	Core set of 11 conserved genes, including JRC, S3H, and a FtsK-like packaging ATPase, found in all NCLDVs; reconstructed ancestor with ~40 genes	[50–53, 107]

Abbreviations: JRC, Jelly-Roll Capsid protein; LTR, Long Terminal Repeat; RdRp, RNA-dependent RNA polymerase; RCRE, Rolling Circle Replication (initiation) Endonuclease; RT, Reverse Transcriptase; S3H, Superfamily 3 Helicase.

### A brief natural history of viral genes

Sequence analysis of viral proteins reveals several categories of virus genes that markedly differ in their provenance. The optimal granularity of classification might be subject to debate but at least 5 classes that can be assorted into three larger categories seem to be readily distinguishable.

Genes with readily detectable homologs in cellular life forms

1. Genes with closely related homologs in cellular organisms (typically, the host of the given virus) present in a narrow group of viruses.

2. Genes that are conserved within a major group of viruses or even several groups and have relatively distant cellular homologs.

Virus-specific genes

3. ORFans, i.e., genes without detectable homologs except, possibly, in closely related viruses.

4. Virus-specific genes that are conserved in a (relatively) broad group of viruses but have no detectable homologs in cellular life forms.

Viral hallmark genes

5. Genes shared by many diverse groups of viruses, with only distant homologs in cellular organisms, and with strong indications of monophyly of all viral members of the respective gene families, – we would like to coin the phrase "viral hallmark genes" to denote these genes that can be viewed as distinguishing characters of the "virus state".

The relative contributions of each of these classes of genes to the gene sets of different viruses strongly depend on the viral genome size which differs by more than three orders of magnitude. Viruses with small genomes, such as most of the RNA viruses, often have only a few genes, the majority of which belong to the hallmark class. By contrast, in viruses with large genomes, e.g., poxviruses, all 5 classes are broadly represented. In order to illustrate the diversity of viral "genomescapes" more concretely, we show in Table 2 the decomposition of the gene sets of three viruses with a small, an intermediate-sized, and a large genomes, respectively, into the 5 classes. Notably, moderate-sized and large genomes of bacteriophages and archaeal viruses are dominated by ORFans that often comprise >80% of the genes in these viruses. Rapidly evolving phage ORFans are thought to supply many, if not most, of the ORFans found in prokaryotic genomes (the lack of detectable sequence conservation notwithstanding), hence playing an important role in evolution of prokaryotes [12].

**Table 2:** Representation of the five classes of viral genes in three selected viruses with small, medium-size and large genomes

	**Genome size (kb)/number of annotated genes**	**Representation of the 5 classes of viral genes (number and brief description)**				
		
		**1. Recent acquisitions from cells**	**2. Ancient acquisitions from cells**	**3. ORFans**	**4. Conserved virus-specific genes**	**5. Hallmark genes**
**Virus**						
**Poliovirus**^**a**^	7.4/10	None	2: a duplication of a chymotrypsin-like protease (3C, 2A)	1: uncharacterized protein (3A)	1: genome-linked protein (VPg)	6: 4 diverged copies of JRC (VP1-4), S3H (3C), RdRp (3D)
***Sulfolobus *turreted icosahedral virus (STIV)**^**b**^	17.6/36	3: two predicted transcription regulators and an uncharacterized protein	5: four predicted transcription regulators and an uncharacterized protein	26: uncharacterized proteins	None	2: JRC, packaging ATPase
**Vaccinia virus**^**c**^	194.7/~200	~48: primarily, proteins involved in virus-host interaction	~36: primarily, proteins involved in genome replication and expression	~24: poorly characterized proteins, possibly, involved in virus-host interactions	~84: primarily, structural components of the virion and some proteins involved in genome expression	5: JRC, S3H/primase, packaging ATPase, DNA polymerase(?)

^a^The classification is based on the analysis described in [87].

^b^The classification is based on the analysis described in [11].

^c^The classification is based on the analysis described in [108] and EVK, unpublished observations; the uncertainty in the number of genes is due to the pseudogenization of varying subsets of genes in different strains of vaccinia virus.

The evolutionary origins of the 5 classes of viral genes are likely to be very different. The least controversial are the two classes of genes with readily detectable homologs from cellular life forms that appear to represent, respectively, relatively recent (class 1) and ancient (class 2) acquisitions from the genomes of the cellular hosts. Where do virus-specific genes come from is a much harder question. In the absence of direct evidence, the default hypothesis, probably, should be that these genes evolved from cellular genes as a result of dramatic acceleration of evolution linked to the emergence of new, virus-specific functions, such that all traces of the ancestral relationships are obliterated. This notion is compatible with the fact that many, probably, most class 4 genes (virus-specific genes conserved within a group of viruses) are virion components (e.g., see the vaccinia virus case in Table 2), a quintessential viral function. The hallmark genes that cross the barriers between extremely diverse virus lineages seem to be of the greatest interest and relevance for the problem of the ultimate origins of viruses, at least, in the context of the long argument we attempt to develop here. Thus, we discuss the distribution among viruses, evolution and significance of these genes in a separate section.

### Viral hallmark genes: beacons of the ancient virus world

Although there are no traceable vertical relationships between large groups of viruses outside the major classes listed in Table 1, a considerable number of genes that encode proteins with key roles in genome replication, expression, and encapsidation are shared by overlapping arrays of seemingly unrelated groups of viruses. As already noted above, some of these widespread viral genes are genuine viral hallmarks that are found in a variety of diverse viruses (although never in all viruses) but not in cellular life forms except as easily recognizable proviruses or mobile elements (Table 3, Fig. 1). The two proteins that are most widely dispersed among viruses are the jelly-roll capsid protein (JRC) [13-15] and the superfamily 3 helicase (S3H) [16]. Each of these proteins crosses the boundary between RNA and DNA viruses and spans an astonishing range of virus groups, from some of the smallest positive-strand RNA viruses to the nucleo-cytoplasmic large DNA viruses (NCLDV), the class of viruses that includes the giant mimivirus (Table 3). Other proteins listed in Table 3 are not as common as JRC or S3H but still form multiple, unexpected connections between groups of viruses that otherwise appear to be unrelated. A case in point is the rolling circle replication (RCR) initiation endonuclease (RCRE) which unites a great variety of small ss and dsDNA replicons, including viruses, plasmids, and transposable elements that reproduce in animals, plants, bacteria, and archaea [17-20]. Notably, a more recent and more careful analysis has shown that the DNA-binding domain of the replication protein of polyoma and papilloma viruses (e.g., the T antigen of SV40) is a derived form of the RCRE that has lost the catalytic amino acid residues [18]. Thus, through this detailed analysis of one of the hallmark proteins, the well-known connection between a variety of small ssDNA-replicons is extended to a group of similar-sized dsDNA-replicons. A similar expansion of the set of viral groups covered by a particular hallmark gene resulted from the detailed analyses of the packaging ATPase and the archaeo-eukaryotic primase (Table 3).

**Table 3:** Proteins encoded by hallmark viral genes

**Protein**	**Function**	**Virus groups**	**Homologs in cellular life forms**	**Comments**	**References**
**Jelly-roll capsid protein (JRC)**	Main capsid subunit of icosahedral virions	Picornaviruses, comoviruses, carmoviruses, dsRNA phage, NCLDV, herpesviruses, adenoviruses, papovaviruses, parvoviruses, icosahedral DNA phages and archaeal viruses	Distinct jelly-roll domains are seen in eukaryotic nucleoplasmins and in protein-protein interaction domains of certain enzymes	Certain icosahedral viruses, such as ssRNA phages and alphaviruses, have unrelated capsid proteins. In poxviruses, the JRC is not a virion protein but forms intermediate structures during virion morphogenesis	[13–15, 53, 54, 109–111]
**Superfamily 3 helicase (S3H)**	Initiation and elongation of genome replication	Picornaviruses, comoviruses, eukaryotic RCR viruses, NCLDV, baculoviruses, some phages (e.g., P4), plasmids, particularly, archaeal ones	S3H is a distinct, deep-branching family of the AAA+ ATPase class	Characteristic fusion with primase in DNA viruses and plasmids	[16, 112]
**Archaeo-eukaryotic DNA primase**	Initiation of genome replication	NCLDV, herpesviruses, baculoviruses, some phages	All viral primases appear to form a clade within the archaeo-eukaryotic primase family	Characteristic fusion with S3H in most NCLDV, some phages, and archaeal plasmids	[18]
**UL9-like superfamily 2 helicase**	Initiation and elongation of genome replication	Herpesviruses, some NCLDV, some phages	Viral UL9-like helicases form a distinct branch in the vast superfamily of DNA and RNA helicases	Fusion with primase in asfarviruses, mimiviruses	[53]
**Rolling-circle replication initiation endonuclease (RCRE)/origin-binding protein**	Initiation of genome replication	Small eukaryotic DNA viruses (parvo-, gemini-, circo-, papova), phages, plasmids, and eukaryotic helitron transposons	No cellular RCRE or papovavirus-type origin-binding protein; however, these proteins have a derived form of the palm domain that is found in the majority of cellular DNA polymerases	Papovaviruses have an inactivated form of RCRE that functions as origin-binding protein	[17–20]
**Packaging ATPase of the FtsK family**	DNA packaging into the virion	NCLDV, adenoviruses, polydnaviruses, some phages (e.g., P9, M13), nematode transposons	A distinct clade in the FtsK/HerA superfamily of P-loop NTPases that includes DNA-pumping ATPases of bacteria and archaea		[113]
**ATPase subunit of terminase**	DNA packaging into the virion	Herpesviruses, tailed phages	The terminases comprise a derived family of P-loop NTPases that is distantly related to Superfamily I/II helicases and AAA+ ATPases		[109, 114]
**RNA-dependent RNA polymerase (RdRp)/reverse transcriptase (RT)**	Replication of RNA genomes	Positive-strand RNA viruses, dsRNA viruses, retroid viruses/elements, possibly, negative-strand RNA viruses	Another major group of palm domains that are distinct from those in DNA polymerases	The RdRps of dsRNA viruses are homologs of positive-strand RNA virus polymerases. The provenance of negative-strand RNA virus RdRp remains uncertain although sequence motif and, especially, structural analysis suggests their derivation from positive-strand RNA virus RdRps	[23–25, 28, 87, 115]

Abbreviations: NCLDV, Nucleo-Cytoplasmic Large DNA Viruses

Replication of positive-strand RNA viruses, dsRNA viruses, negative-strand RNA viruses, and retroid viruses/elements is catalyzed by another idiosyncratic class of viral enzymes, RNA-dependent RNA polymerases (RdRp) and reverse transcriptases (RT). The positive-strand RNA virus RdRp and the RT form a monophyletic cluster within the vast class of the so-called palm-domains that are characteristic of numerous polymerases [21-24]. The RdRps of dsRNA viruses and negative-strand RNA viruses are likely to be highly diverged derivatives of the same polymerase domain, an old conjecture [24-26] that has been vindicated by the recent determination of the structure of a dsRNA bacteriophage RdRp [27,28].

The palm domain is likely to be the primordial protein polymerase that emerged from the RNA world where nucleotide polymerization was catalyzed by ribozymes [29]. This is supported not only by the wide spread of this domain in modern life forms but also by the structural and, by inference, evolutionary link between the palm domain and the RNA-recognition-motif (RRM) domain, an ancient RNA-binding domain that might have, initially, facilitated replication of ribozymes [30]. The palm-domain RdRps and RTs are excluded from the regular life cycles of cellular life forms, although most eukaryotic genomes encompass numerous copies of RT-containing retroelements, and prokaryotes have some such elements as well [31,32]. These elements, however, are selfish and, from the evolutionary standpoint, virus-like. The most notable incursion of an RT into the cellular domain is the catalytic subunit of the eukaryotic telomerase, the essential enzyme that is involved in the replication of chromosome ends [33,34].

The list of viral hallmark genes given in Table 3 is a conservative one. There well might be other genes that merit the hallmark status but for which clear evidence is hard to come up with. An important case in point is the B-family DNA polymerase that is the main replication enzyme of numerous dsDNA viruses of bacteria and eukaryotes. Homologs of these DNA polymerases are found in all archaeal and eukaryotic genomes, so that monophyly of all viral polymerases does not seem to be demonstrable in phylogenetic analyses [35,36]. However, this potentially could be explained by relatively (with respect to cellular homologs) fast evolution of the polymerases in various viral lineages, which would obscure their common origin. Furthermore, monophyly of the polymerases of all viruses that employ a protein-primed mechanism of dsDNA replication (animal adenoviruses and tailed phages like PRD1 or φ29) has been claimed [37]. Thus, although this currently cannot be shown convincingly, it seems possible (and, as discussed below, even likely) that the DNA polymerase is a viral hallmark gene in disguise. More generally, further sequencing of viral genomes, combined with comprehensive comparative analysis, might reveal additional genes that, despite relatively limited spread among viral lineages, will qualify as hallmark genes.

The combination of features of viral hallmark proteins is highly unusual and demands an evolutionary explanation. Indeed, the hallmark genes are, without exception, responsible for essential, central aspects of the viral life cycles, including genome replication, virion formation, and packaging of the genome DNA into the virion (Table 3). These genes span sets of extremely diverse classes of viruses, often possessing different reproduction strategies and differing by three orders of magnitude in genome size. Finally, all viral hallmark genes have remote homologs in cellular life forms (Table 3) but the viral versions appear to be monophyletic.

Three hypotheses on the origin of viral hallmark genes immediately come to mind. The first possibility is that the notion of hallmark virus proteins is based on an artifact. The argument, that is commonly invoked in discussions of unexpected patterns of homologous relationships and could well be waged against the notion of viral hallmark proteins, is that genuine orthologs of these proteins (direct evolutionary counterparts, typically, with the same function) actually do exist in cellular life forms but are not detectable due to rapid sequence divergence between viral and cellular proteins. However, this reasoning does not seem to survive closer scrutiny. Firstly, the conservation of the hallmark proteins in extremely diverse classes of viruses with widely different replication/expression strategies (Table 3) but not in cellular life forms is hardly compatible with the rapid divergence interpretation. Indeed, for this to be the case, acceleration of evolution of the hallmark genes in diverse classes of viruses should occur in such a manner that the similarity between viral proteins survived, whereas the similarity between viral proteins and their hypothetical cellular orthologs vanished. Parallel conservation of hallmark protein sequences might be perceived in the case of structural protein, such as JRC, but is hardly imaginable for proteins involved in replication of structurally very different genomes, such as S3H that is conserved among viruses with RNA, ssDNA, and dsDNA genomes. Furthermore, for most of the hallmark proteins, distant and functionally distinct homologs from cellular organisms are detectable (S3H and viral RNA-dependent polymerases are the primary examples) which makes the existence of elusive orthologs extremely unlikely.

The other two hypotheses accept the hallmark viral proteins as reality but offer contrasting evolutionary scenarios to account for their existence and spread.

1. One hypothesis would posit that the hallmark genes comprise the heritage of a "last universal common ancestor of viruses" (LUCAV). This scenario implies that, despite all evidence to the contrary (see above) all extant viruses are monophyletic, although their subsequent evolution involved massive gene loss in some lineages as well as extensive acquisition of new genes from the hosts in others.

2. By contrast, under the hypothesis of polyphyletic origin of viruses, the spread of the hallmark genes across the range of virus groups could be explained by horizontal gene transfer (HGT).

Upon closer inspection, none of these hypotheses seems to be a viable general explanation for the existence and distribution of the viral hallmark genes. Indeed, the relatively small number and the mosaic spread of the hallmark genes (Table 3) do not seem to be conducive to the LUCAV notion although it is apparent that a great number of diverse viruses, if not all of them, share some common history. Conversely, the extremely distant similarity between the hallmark proteins from diverse virus groups with dramatically different replication strategies is poorly compatible with an HGT scenario.

Here, we outline a scenario of virus origin and evolution that does not involve a LUCAV but integrates aspects of the common origin and HGT hypotheses and is naturally linked to specific models of evolution of cells. The simplest explanation of the fact that the hallmark proteins involved in viral replication and virion formation are present in a broad variety of viruses but not in any cellular life forms is that the latter never had these genes in the first place. The alternative that we consider most likely is that the hallmark genes antedate cells and descend directly from a primordial gene pool. It is thought that, in such a primordial pool, selection would act primarily on functions directly involved in replication [38,39] which is compatible with the properties of the majority of hallmark genes (Table 3). Given the spread of the hallmark genes among numerous groups of dramatically different viruses, a crucial corollary is that the major lineages of viruses themselves derive from the same, precellular stage of evolution. This corollary serves as the foundation for the concept of an ancient Virus World, which we envisage as an uninterrupted flow of genetic information through an enormous variety of selfish elements, from the precellular stage of evolution to this day; we discuss the Virus World in the rest of this article.

### Conflicting concepts of virus origin and evolution and the inextricable link between evolution of viruses and cells

Before we discuss the full scope of the emerging concept of the origin of viruses from the precellular gene pool, it is necessary to briefly examine the existing trains of thought on virus origin and evolution. Traditionally, these ideas have revolved around three themes: i) origin of viruses from primordial genetic elements, ii) degeneration of unicellular organisms to the virus state, and iii) "escaped genes" hypotheses deriving viruses from genes of cellular organisms that have switched to the selfish mode of reproduction (reviewed in [40-45]) (Table 4). Historically, it is remarkable that Felix d'Herelle, the discoverer of bacteriophages and one of the founders of virology, proposed that phages might be evolutionary precursors of cells as early as 1922 [46]. Furthermore, in J.B.S. Haldane's 1928 classic on the origin of life [47], an early, "viral" stage of evolution is considered as an integral part of the proposed scenario for the emergence of the first life forms from the primary soup (we revisit Haldane's prescient speculation toward the end of this article). However, the "primordial" hypothesis is habitually dismissed on the grounds that all extant viruses are intracellular parasites, so viruses could not exist before the emergence of modern-type cells although antiquity of viruses has been propounded based on the lack of cellular homologs for many virus genes [43,48-51]. By contrast, the high prevalence of host-related genes (as opposed to virus-specific genes) in many viruses (particularly, those with large genomes) might be construed as support for the "escaped genes" or even the "cell degeneration" hypotheses.

**Table 4:** Major concepts in virus evolution

**Concept**	**Principal message**	**References**	**Brief critique/comment**
Cell degeneration model of virus origin	Viruses, at least complex ones, evolved as a result of degeneration of cells, perhaps, through a stage of intracellular parasites	[40, 43, 45, 50]	This route of virus evolution appears to be inconsistent with the results of viral comparative genomic, in particular, the prominence of genes without cellular counterparts in the conserved cores of viral genomes
Escaped-genes model of virus origin	Viruses evolved from within cells, through autonomization of the appropriate genes, e.g., those coding for polymerases	[40, 43, 45, 55]	Similarly, this model lacks support from virus genome comparison
Origin of viruses from a primordial gene pool	Viruses are direct descendants of primordial genetic elements	[40, 43, 87]	Generally, this appears to be the most plausible path for the origin of viruses. However, non-trivial conceptual development is required, given that viruses are intracellular parasites and, technically, could not precede cells during evolution
An ancient lineage of viruses spanning the three domains of cellular life	The presence of JRC in a variety of groups of DNA viruses is taken as evidence of the existence of an ancient lineage of viruses infecting all three domains of cellular life	[13–15]	This concept capitalizes on a truly remarkable observation of the near ubiquity of JRC in viruses. However, inferring an ancient lineage of viruses on the basis of the conservation of a single protein smacks of essentialism and does little to explain the trajectories of most other virus-specific and virus hallmark genes. Besides, this concept does not specify the cellular context in which the ancient virus lineage might have emerged
Three DNA viruses to replicate genomes of RNA cells	The hypothesis postulates that at least three major lineages of RNA viruses emerged by the escaped-genes route from RNA-based progenitors of archaea, bacteria and eukaryotes. These ancient RNA viruses are thought to have given rise to three independent lineages of DNA viruses that imparted DNA replication onto their cellular hosts	[49, 55]	This concept is based on important general notions of the ancient origin of viruses and their major role in evolution of cells. However, the specific model of Forterre appears to be critically flawed as it stems from a model of cellular evolution that appears not to be defendable (see text)

The existence of the hallmark virus genes seems to effectively falsify both the cell degeneration and the escaped-genes concepts of viral evolution. With regard to the cell degeneration hypothesis, let us consider the NCLDV, the class of large viruses to which the cell degeneration concept might most readily apply and, indeed, was, in the wake of the discovery of the giant mimivirus [50-52]. Among the 11 signature genes that are shared by all NCLDVs ([53] and Table 1), three crucial ones (JRC, S3H, and the packaging ATPase) are virus hallmark genes. A clear inference is that even the simplest, ancestral NCLDV would not be functional without these genes. However, cellular derivation of this ancestral NCLDV would have to invoke decidedly non-parsimonious, ad hoc scenarios, such as concerted loss of all hallmark genes from all known cellular life forms or their derivation from an extinct major lineage of cell evolution. The same line of logic essentially refutes the escaped genes concept inasmuch as the hallmark genes had no cellular "home" to escape from. Again, to save "escaped genes", an extinct cellular domain would have to be postulated.

Two recent conceptual developments in the study of origin and evolution of viruses deserve special attention (Table 4; see also discussion below). First, Bamford and coworkers [13-15] and, independently, Johnson and coworkers [54] capitalized on the conservation of the structure of the jelly-roll capsid protein in a wide variety of viruses to propose the idea of an ancient virus lineage spanning all three domains of cellular life (archaea, bacteria, and eukaryotes). Second, Forterre presented an elaborate scheme of virus-cell coevolution from the earliest stages of life's evolution. Under this concept, viruses emerged independently within three lineages of RNA-based cells (the progenitors of archaea, bacteria, and eukaryotes) and "invented" DNA replication that was subsequently captured from different viruses by each type of host cells, in three independent transitions to DNA genomes [43,55].

A crucial, even if fairly obvious, aspect of viral evolution is that it is inextricably linked to the evolution of the hosts which, when traced back to the earliest stages of life's evolution, attests to the necessity to consider scenarios of virus origins in conjunction with models for the origin and early evolution of cells. This dramatically ups the ante for the study of virus evolution, bringing it to the center stage of evolutionary biology [56,57]. Therein, however, seem to lie some of the major problems encountered by the current hypotheses of virus evolution (Table 4). The concept of an ancient virus lineage advocated by Bamford and coworkers, in addition to being based on the broad spread of a single gene (JRC), which is construed as the "self" of a virus [13], simply leaves a glaring gap as the connection between viral and cellular evolution is not considered such that it remains unclear in what kind of cellular environment the early evolution of the primordial viral lineage took place.

By contrast, Forterre's concept is embedded within a specific scenario of cellular evolution, and we believe that this is the valid approach to the analysis of the origins and evolution of viruses and cells – the only chance to achieve understanding of these difficult problems is to consider them in conjunction. However, the specific scenario of cellular evolution favored by Forterre appears to be poorly compatible with the results of comparative genomics, thus compromising the model of virus evolution associated with it. Indeed, Forterre considers three lineages of RNA-cells evolving from the Last Universal Common Ancestor (LUCA) of the known life forms and giving rise to bacteria, archaea, and eukaryotes, with the transition to DNA-cells mediated by domain-specific viruses. This scenario, while complete with respect to the evolution of both cells and viruses, encounters at least three major, probably, insurmountable problems. First, it is dubious at best that the combination of complexity and genetic stability required of an even a minimal cell – a complement of a few hundred genes that is accurately transmitted over many cellular generations – is attainable with an RNA genome. Indeed, given the inherent instability of RNA, such a genome would have to consist of several hundred RNA molecules. Accurate partitioning of this multipartite genome between daughter cells would require an RNA segregation system of unprecedented precision; thus, faithful vertical inheritance of the genome is hardly imaginable. Stochastic segregation of RNA segments in a polyploid cell might seem to be a straightforward solution to this problem but a quick calculation shows that this is unfeasible. Indeed, if the probability of a the two daughter RNA segments being segregated into the two daughter cells is 1\2, then the probability that, say, 100 RNA segments in a reasonable multipartite genome are all correctly segregated (i.e., none of the segments is missing in either of the daughter cells) is (1/2)^100 ^≈ 10^-30^. Thus, ploidy of ~10^30 ^would be required for accurate genome segregation; in other words, the RNA cell would have to contain many tons of RNA. Thus, it is much more likely that the first fully-fledged cells already had DNA genomes resembling (even if quantitatively simpler than) those of modern archaea and bacteria. Forterre's proposal is based on the well-known observation that the DNA replication systems of archaea and bacteria are, largely, unrelated, making it unlikely that LUCA had a DNA genome [58-60]. We believe that a far more plausible implication of the disparity of DNA replication machineries in archaea and eukaryotes is a non-cellular LUCA, a hypothesis that finds crucial support in the fact that the membrane lipids and membrane biogenesis systems, as well as cell walls, are also distinct and, largely, unrelated in archaea and bacteria [39,61-63].

For efforts on reconstructing the non-cellular LUCA, a key guiding principle is that, although modern-type cells, presumably, did not exist at this stage of evolution, some form of compartmentalization was required to ensure concentrations of substrates and genetic elements sufficient for effective replication and, consequently, evolution. Furthermore, as discussed in greater detail below, compartmentalization is a necessary condition of selection among evolving ensembles of genetic elements. Following this principle, a specific model has been elaborated under which early evolution, from abiogenic syntheses of complex organic molecules to the emergence of archaeal and bacterial cells, unraveled within networks of inorganic compartments that are found at hydrothermal vents and consists, primarily, of iron sulfide [39,63]. Here, in order to be concrete, we attempt a reconstruction of the earliest events in the evolution of primordial virus-like entities within the framework of this model although our general conclusions do not seem to hinge on any particular model of organization of the ancient, precellular life.

Second, the model by viral overtake meets a stumbling block: if three different viruses brought the three distinct DNA replication machineries into the three cell lineages, why have not the hallmark genes that are essential for viral DNA replication, in particular S3H and the viral-type primase, entered the genomes of any of those cellular lineages? One could argue that, in each case, an unusual virus, not carrying any of these hallmark genes, was involved, but this happening three times independently stretches credulity.

The third major issue that is ignored in Forterre's hypothesis and similar, three-domain scenarios of cellular evolution [64,65], is the readily demonstrable relationship between eukaryotes and the two prokaryotic domains. This relationship follows the divide between the two principal functional categories of genes in the eukaryotic cell, the informational genes that are, almost invariably, most closely related to archaeal homologs, and the operational genes that are of bacterial provenance [66-68]. By far the simplest, most parsimonious explanation of this dichotomy is that the eukaryotic cell emerged as a result of a symbiosis between an archaeon and a bacterium. Given the overwhelming evidence of the origin of mitochondria and related organelles (hydrogenosomes and mitosomes) from α-proteobacteria, the nature of the partners and the direction of the symbiosis appear clear: an α-proteobacterium invaded an archaeal cell [69-71]. In addition to the genomic evidence, there is a clear biochemical rationale for the symbiosis to actually comprise the onset of eukaryogenesis such that the invading α-proteobacterium became an anaerobic symbiont that originally supplied hydrogen to the methanogenic, archaeal host and subsequently gave rise to aerobic mitochondria [71]. The massive transfer of the symbiont's genes to the host genome gave rise to the mosaic provenance of the eukaryotic genomes. The argument against the symbiotic models of eukaryogenesis and for three-domain models stems primarily from the existence of numerous eukaryote-specific proteins (ESPs) without readily detectable prokaryotic homologs [64,72,73]. However, the abundance of ESPs hardly can be taken as evidence of a third domain of life, distinct from archaea and bacteria, and comprising the pre-symbiosis eukaryotic line of descent. First, although many of the ESPs are proteins with important biological functions, they do not belong to the core of either the informational or the metabolic proteins repertoires of eukaryotes that are of archaeal and bacterial descent, respectively. Second, more often than not, prokaryotic homologs of an apparent ESP can be identified by using sensitive techniques of protein motif analysis and, particularly, structural comparison [74]. Thus, it seems likely that many, if not most, ESPs are cases of accelerated evolution in eukaryotes which accompanies the emergence of novel, eukaryote-specific functional systems, such as the cytoskeleton and ubiquitin signaling. The prevalence and nature of "true" eukaryotic innovations, i.e., proteins that evolved through processes other than descent with (perhaps, radical) modification from archaeal or bacterial proteins, remain to be characterized. Clearly, however, even if such novelties turn out to be numerically prominent, they are, typically, involved in ancillary functions and hardly can be construed as the heritage of a distinct primary line of cell evolution [74].

In the rest of this article, we consider evolution of viruses in conjunction with these two central concepts of cellular evolution: i) the existence of an early non-cellular but confined stage in life's evolution, that probably encompassed LUCA, and ii) the origin of the eukaryotic cell as a result of fusion of an archaeon and a bacterium. We argue that the scenario of virus origin and evolution informed by comparative genomics forms a coherent whole with these notions of early cell evolution and provides supportive feedback for them.

### The primordial gene pool: the crucible of the major virus lineages

**Figure 2:**
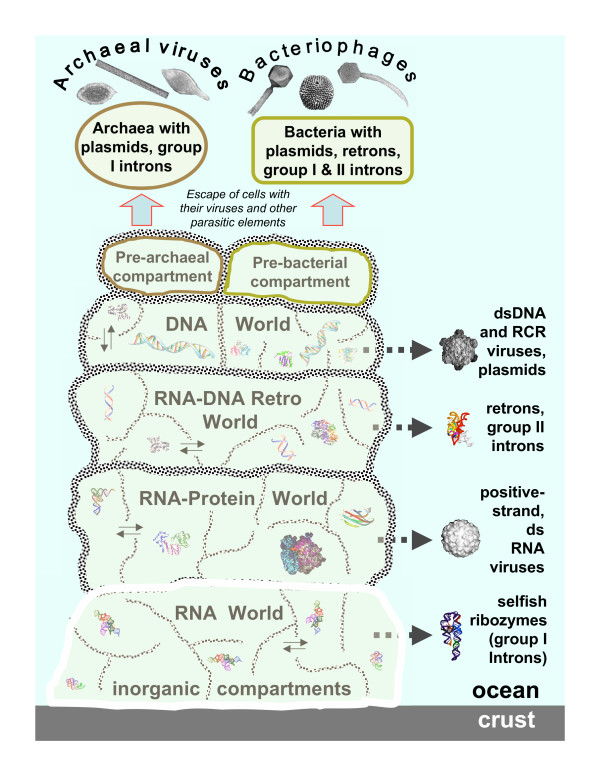
**Evolution of the virus world: origin of the main lineages from the primordial gene pool**. Characteristic images of RNA and protein structures are shown for each postulated stage of evolution, and characteristic virion images are shown for the emerging classes of viruses. Thin arrows show the postulated movement of genetic pools between inorganic compartments. Block arrows show the origin of different classes of viruses at different stages of pre-cellular evolution.

Taken together, all these lines of evidence and reasoning suggest that the principal classes of prokaryotic viruses, including positive-strand RNA viruses, retroid elements, and several groups of DNA viruses, emerged within the primordial genetic pool where mixing and matching of diverse genetic elements was incomparably more extensive than it is in any modern biological community [39,75] (Fig. 2).

As indicated above, we present a scenario of viral origins under the recent model of the emergence of cells and genomes within networks of inorganic compartments [39,63]. These compartments are envisaged being inhabited by diverse populations of genetic elements, initially, segments of self-replicating RNA, subsequently, larger and more complex RNA molecules encompassing one or a few protein-coding genes, and later yet, also DNA segments of gradually increasing size. Thus, early life forms, including LUCA, are perceived as ensembles of genetic elements inhabiting a system of inorganic compartments. This model explains the lack of homology between the membranes, membrane biogenesis systems, and the DNA replication machineries of archaea and bacteria by delineating a LUCA that had neither a membrane nor DNA replication. The model also outlines the processes that might have enabled selection and evolutionary change in such a system. Under this scenario, a transition gradually took place from selection at the level of individual genetic elements to selection for ensembles of such elements encoding both enzymes directly involved in replication and proteins responsible for accessory functions, such as translation and nucleic acid precursor synthesis. From selection for gene ensembles, there is a direct path to selection for compartment contents such that compartments sustaining rapid replication of genetic elements would infect adjacent compartment and, effectively, propagate their "genomes" [39].

This model implies that, at the early stages of evolution, including the LUCA stage, the entire genetic system was, in a sense, "virus-like". Initially, all RNA segments in the population would be completely selfish, and there would be no distinction between parasitic, "viral" elements and those that would give rise to genomes of cellular life forms. However, this distinction would emerge as soon as the first "selfish cooperatives" – relatively stable ensembles of co-inherited genetic elements [39] – evolve. Inevitably, the selfish cooperatives would harbor parasitic genetic elements that would divert the resources of the ensemble for their own replication. The separation of parasitic elements from cooperators would be cemented by the emergence of physical linkage of the latter: genes that encode components of the replication machinery and are physically linked to genes for accessory functions would be locked into ensembles of cooperators, whereas solitary genes (or small gene arrays) encoding replication functions would occupy the parasitic niche. From a complementary standpoint, within the inorganic compartment model, the distinction between the progenitors of viruses and the ancestors of cellular life forms may be described as one between primary infectious agents, that routinely moved between compartments, and increasingly complex and "sedentary" ensembles of selfish cooperators that would spill from one compartment into another on much rarer occasions.

Like other models of the early stages of evolution of biological complexity, this scenario faces the problem of takeover by selfish elements [76-78]. If the primordial parasites become too aggressive, they would kill off their hosts within a compartment and could survive only by infecting a new compartment. Devastating "epidemics" sweeping through entire networks and eventually eliminating all their content are imaginable, and indeed, this might have been the fate of many, if not most, primordial "organisms". The evolution of those primordial life forms that survived would involve, firstly, emergence of temperate virus-like agents that do not kill the host, and secondly, early invention of primitive defense mechanisms, likely, based on RNA interference (RNAi). The ubiquity of both temperate selfish elements and RNAi-based defense systems [79-81] in all life forms is compatible with the origin of these phenomena at a very early stage of evolution.

From the very beginning, the parasites would have different levels of genome complexity, from the minimal replicable unit, like in viroids, to more complex entities that might encode one or more proteins facilitating their own replication. Capsid proteins, in particular, JRC were the crucial innovation that might have led from virus-like genetic elements to entities resembling true viruses (as long as viruses are defined as intracellular parasites, we are not entitled to speak of viruses evolving prior to the appearance of membrane-bounded cells; however, this is a semantic problem only). Under this model, the advantage conferred on a selfish genetic element by encapsidation is obvious: stabilization of the genome and increased likelihood of transfer between compartments and even between different networks of compartments. Given this advantage of the capsid and the extensive reassortment and recombination that is thought to have reigned in the primordial gene pool, it is not surprising that different genes that could contribute to virus replication, such as polymerases, helicases, ATPases, and nucleases, combined with the JRC gene on multiple occasions, and several such combinations were propagated by selection to give rise to distinct viral lineages. Alternative capsid proteins that form helical capsids in diverse RNA and DNA viruses [82] might have evolved already at this early stage of evolution. However, the capsid is by no means a pre-requisite of evolutionary success for selfish genetic elements. Indeed, virus-like entities, such as the prokaryotic retroelements (retrons and group II introns) and various small plasmids, have maintained the capsid-less, selfish life style from their inferred emergence within the primordial gene pool to this day.

Thus, under this scenario, virus-like entities are older than typical, modern-type cells: agents resembling modern viruses, some of them even with capsids, existed before the emergence of membrane-bounded cells with large dsDNA genomes. Moreover, these agents would, effectively, have a virus-like life style because the primordial life forms are perceived as non-cellular but compartmentalized and would support ancient virus-like agents moving between compartments much like modern cells support viruses. The origin of ancient virus-like agents, the ancestors of the major classes of modern viruses, would follow the stages of evolution of genetic systems: RNA-only selfish agents resembling group I introns or viroids might have emerged in the primitive RNA world. The RNA-protein world begot RNA viruses, the postulated stage of an RT-based, mixed RNA-DNA system spawned retroid elements, and the DNA stage yielded several lineages of DNA viruses (Fig. 2). It appears likely that the primordial gene pool harbored an extraordinary variety of virus-like entities. When archaeal and bacterial cells escaped the compartment networks, they would carry with them only a fraction of these viruses that subsequently evolved in the cellular context to produce the modern diversity of the virus world.

General considerations on the course of evolution from the RNA world to modern-type systems, together with the spread of different virus classes among modern life forms (Table 1 and Fig. 1), suggest that the following classes of virus-like selfish elements emerged from the primordial gene pool and infected the first bacteria and/or archaea (Fig. 2): i) RNA-only elements, such as group I introns, ii) positive-strand RNA viruses (and, possibly, dsRNA viruses as well), iii) retroid elements similar to prokaryotic retrons and group II introns, iv) small RCR replicons, v) at least one, and probably, more groups of larger, dsDNA viruses ancestral to the order Caudovirales (tailed phages). Incidentally, the logic of this inference suggests that the dsDNA viruses emerging from the primordial gene pool already possessed DNA polymerases and, accordingly, that the DNA polymerase is a true viral hallmark gene, notwithstanding the difficulty in obtaining strong evidence of this (see above).

To conclude the discussion of viral origin from the primordial genetic pool, we would like to emphasize, once again, the tight coupling between the earliest stages of viral and cellular evolution. Indeed, the presence of viral hallmark genes in extremely diverse groups of viruses, combined with a variety of other genes, constitutes strong evidence of extensive reassortment/recombination associated with the origin of viruses, which is best compatible with a primordial gene pool with rampant gene mixing and matching. Thus, viral comparative genomics seems to provide substantial support for the non-cellular model of early evolution. However, it should be noticed that the virus world concept does not necessarily require a non-cellular LUCA; the concept would survive even if LUCA was, actually, a cell. What is germane to our model is the existence of an advanced pre-cellular stage of evolution at which substantial genetic diversity was already attained; whether LUCA existed at that or at a later stage, while an extremely important and intriguing issue in itself, is not central to our argument.

### Origin of eukaryotic viruses: the second melting pot of viral evolution

Origin of the eukaryotic viruses is a distinct and fascinating problem. Two features of eukaryotic viruses are most relevant for this discussion:

i) with the sole exception of large dsDNA viruses, all major classes of viruses display greater diversity in eukaryotes than in prokaryotes;

ii) although eukaryotic viruses share a substantial number of genes with bacteriophages and other selfish genetic elements of prokaryotes, the relationships between prokaryotic and eukaryotic viral genomes are always complex, to the extent that direct, vertical links between specific groups of eukaryotic and prokaryotic viruses often are not traceable (Table 5).

**Table 5:** Evolutionary connections between prokaryotic and eukaryotic viruses and related selfish genetic elements

**Lineages of eukaryotic viruses**	**Lineages of prokaryotic viruses**	**Shared genes**	**Type of relationships**	**References**
**Positive-strand RNA viruses**	Positive-strand RNA bacteriophages (MS2, etc)	RdRp	Possible direct vertical link (monophyly) although capsid proteins of RNA phages are unrelated to those of eukaryotic viruses	[87]
**Retroid viruses and elements**	Retrons, group II introns	RT	Possible direct vertical relationship although eukaryotic viruses/elements have many additional genes including proteases and virion components; none of the prokaryotic elements have capsids.	[32, 103, 104]
**Parvoviruses, papovaviruses, circoviruses, geminiviruses, helitron transposons**	Small DNA bacteriophages (e.g., φX174) and plasmids	RCRE	Generic evolutionary relationship linked to the common mode of replication	[17–19]
**Adenoviruses**	Tailed bacteriophages with genome-linked terminal proteins (e.g., PRD1)	JRC, DNA polymerase, terminal protein, packaging ATPase	Possible direct relationship suggested by the coherent set of conserved proteins	[116]
**Herpesviruses**	Tailed bacteriophages	JRC, large terminase subunit, UL9 helicase, DNA polymerase, assemblin (virion morphogenetic protease)	Possible direct relationship suggested by the coherent set of conserved proteins. However, a more complex relationship with different phages might be more likely	[109, 117]
**Nucleo-Cytoplasmic Large DNA viruses (NCLDV)**	Tailed bacteriophages, plasmids	JRC, S3H, primase, packaging ATPase, Holliday junction resolvase, helicases	Complex relationships with different groups of phages and plasmids; in particular, the fusion primase-S3H protein most closely resembles a homolog from archaeal plasmids.	[53, 107]

**Figure 3:**
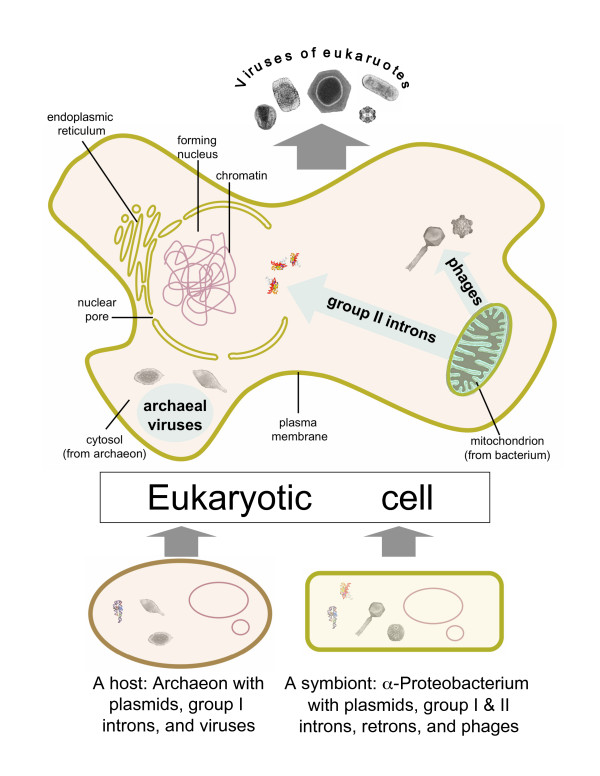
**The second melting pot of virus evolution: origin of eukaryotic viruses**. Characteristic images of archaeal, bacterial, and eukaryotic viruses are shown.

This implicates the emerging eukaryotic cell as a second, after the primordial gene pool, melting pot of virus evolution, in which extensive mixing and matching of viral and cellular genes molded a new domain of the virus world (Fig. 3).

As discussed above, it seems most likely that the eukaryotic cell emerged via an archaeo-bacterial fusion. Specifically, the most parsimonious scenario seems to be that engulfment of an α-proteobacterium by a methanogenic archaeon, leading to the origin of the first, initially, anaerobic mitochondriate cell, had been the starting point of eukaryogenesis [70,71]. Conceivably, mitochondrial symbiogenesis set off a dramatic series of events that led to the emergence of the eukaryotic cell in a relatively short time. The mitochondria, probably, have spawned the invasion of group II introns into the host genes; this onslaught of introns could have been the driving force behind the origin of the nucleus [70]. The mitochondria also donated numerous genes that integrated into the host genome, including genes coding for components of the essential organelles of the eukaryotic cells, such as the endoplasmic reticulum, the nucleus, and the bacterial-type plasma membrane that displaced the original archaeal membrane [66,83]. Furthermore, several bacteriophage genes, in all likelihood, from the mitochondrial endosymbiont's phages, have been recruited for replication and expression of the mitochondrial genome [84,85]. Even more notably, the catalytic subunit of the telomerase, the pan-eukaryotic enzyme that is essential for the replication of linear chromosomes appears to have evolved from a prokaryotic retroid element [33,34]. Thus, prokaryotic selfish elements apparently supplied at least one key component of the eukaryotic cell replication machinery.

The relationships between bacteriophage genes and those of eukaryotic viruses (Table 5) imply that the mitochondria and, possibly, other, transient endosymbionts also contributed many genes from their phage gene pool to the emerging eukaryotic viruses [53,85]. Based on the current knowledge of bacterial and archaeal virus genomics, bacteriophages of the endosymbiont(s) played a much greater role in the origin of eukaryotic viruses than archaeal selfish elements (Table 5). In particular, eukaryotic RNA viruses apparently could have been derived only from the respective phages inasmuch as no archaeal RNA viruses have been so far discovered. Retroid elements are also most characteristic of bacteria, α-proteobacteria in particular (those few retroids that have been discovered in isolated archaeal species are thought to be the results of relatively recent HGT from bacteria), such that the remarkable proliferation of these elements in eukaryotes, most likely, was spawned by mitochondrial endosymbiosis [86]. The case of DNA viruses is more complicated because both bacteriophages and archaeal viruses and plasmids share genes with eukaryotic DNA viruses (Table 5). Thus, different groups of eukaryotic DNA viruses might have inherited genes from either bacteriophages or archaeal viruses (and other selfish elements), or a mixture thereof.

The pivotal contribution of prokaryotic viruses to the origin of eukaryotic viral genomes appears to be as strongly supported by the conservation of the hallmark genes across most of the virus world as the original emergence of viruses from the primordial gene pool. Indeed, since the viral hallmark genes have never become integral parts of the cellular gene pool, the only source from which eukaryotic viruses could inherit these essential genes was the gene pool of prokaryotic viruses, plasmids, and other selfish elements. Obviously, while the hallmark genes comprise a large fraction of the gene complement in small viruses, such as most of the RNA viruses and retroid viruses/elements, in large DNA viruses, these genes form only a small genomic kernel (see Table 2 and discussion above). Accordingly, as far as small viruses are concerned, the notion of direct evolutionary derivation of eukaryotic viruses/elements from prokaryotic counterparts might be justified. In particular, although positive-strand RNA viruses of eukaryotes share only one gene, the RdRp, with RNA bacteriophages, this gene occupies more than half of the genome coding capacity in the phages and the smallest eukaryotic viruses, and its product is either the sole virus-specific component of the replication machinery of the respective viruses or, at least, constitutes its catalytic core. This prominence of the conserved RdRp gene supports the view that bacterial and eukaryotic RNA viruses are linked by a direct, vertical relationship. The same logic could apply to retroid viruses/elements, where the common denominator is the RT, and to RCR elements (viruses and plasmids) that are unified by the presence of the RCRE. However, even small viruses of eukaryotes carry signs of recombination bringing together genes from different sources, including hallmark genes that are not found next to each other in prokaryotic viruses. An obvious case in point is the juxtaposition of the RdRp gene with the genes for JRC and/or S3H in eukaryotic positive-strand RNA viruses [87]. The latter two genes are not seen in RNA bacteriophages, suggesting, however counter-intuitively, that DNA phages (and plasmids, in the case of S3H), many of which possess genes for these proteins, made major contributions to the evolution of eukaryotic RNA viruses. Even more dramatically, eukaryotic retroid viruses possess, in addition to the RT, a diverse array of genes of apparently different provenance, including RNAse H, a ubiquitous cellular enzyme whose exact origin in retroviruses is hard to infer [88], the integrase, probably derived from bacterial transposons [89,90], the protease of likely mitochondrial origin [91], and the capsid/envelope proteins whose ancestry remains uncertain. Thus, the conclusion is inevitable that eukaryotic retroviruses and retrotransposons evolved through a complex series of recombination events between selfish elements of diverse origins [88] and, possibly, genes of cellular origin as well.

The large dsDNA viruses of eukaryotes are a different lot altogether, with several classes of genes of different origins and ages (see discussion above and Table 2). The conserved core in each lineage of large eukaryotic dsDNA viruses consists of a small set of hallmark genes along with some less common genes shared with bacteriophages, other bacterial selfish elements, and/or bacteria, and a few genes that appear to be unique to eukaryotes and have been acquired by the viruses at an early stage of eukaryogenesis. In addition to the core genes, each group of large viruses acquired numerous genes, mostly, from eukaryotes but, in some cases, also from bacteria; often, these genes play roles in virus-host interactions, and some of them seem to have been scavenged by viruses relatively recently (Table 2; [53,92]). Large viruses also have many "unique", lineage-specific genes the provenance of which remains uncertain. This "multilayer" genome organization of large viruses, along with the observations that even the hallmark genes often connect the same group of eukaryotic viruses to different prokaryotic selfish elements (Table 5), does not seem to support direct derivation of these viruses from individual phages.

The above data and considerations clearly point to a great amount of genomic perturbation during the emergence of eukaryotic viruses. However, from a more general perspective, one must remember the Darwinian principle that evolution can only proceed via a succession of manageable steps each of which is associated with an increase in fitness. Hence the emergence of eukaryotic viruses involves an apparent paradox: an uninterrupted succession of viruses must connect the prokaryotic and eukaryotic domains of the virus world, even if, in most cases, this succession cannot be unequivocally identified in terms of shared genes. From this standpoint, the notion that even a single shared protein, such as the JRC, represents a lineage of virus evolution [13-15] appears relevant. However, other viral hallmark genes, as well as less widespread genes shared by phages and eukaryotic viruses (Table 5), comprise additional lines of descent connecting the prokaryotic domain of the virus world with the eukaryotic domain. These lines of viral gene evolution form multiple intersections in the melting pot of eukaryogenesis: the path between the two domains was not smooth but rather involved drastic remolding of the viral genomes. Certainly, this conclusion must be taken with the caveat that the available bacteriophage genomes account for but a tiny slice of the enormous phage diversity, so it is impossible to rule out that direct progenitors of the main groups of eukaryotic viruses are lurking in the unexplored parts of the phage domain. Still, given the numerous connections at the level of individual genes between prokaryotic and eukaryotic viruses discovered so far and the lack of wholesale genomic links, we venture to extrapolate that the future, massive sequencing of phage genomes brings more of the same: we will be able to identify phage roots of additional eukaryotic viral genes but (in most cases) not genome-wide vertical relationships.

As pointed out above, the representation of different genome strategies are dramatically different between prokaryotic and eukaryotic viruses, the foremost distinction being the higher prevalence of DNA viruses in prokaryotes contrasted by the preponderance of RNA viruses in eukaryotes (Fig. 1). The cause(s) of this disparity is a major puzzle in virology but consideration of the differences in the cellular organization of prokaryotes and eukaryotes might offer a clue. Conceivably, the emergence of the nucleus substantially changed the distribution of niches available for viruses by sequestering the machineries for host cell DNA replication, repair, and transcription within the nucleus and hence hampering the exploitation of these systems by DNA viruses, a mode of reproduction that is readily accessible to bacteriophages. Hence the relative decline in DNA virus diversity in eukaryotes and the massive spread of RNA viruses into the freed niches.

Thus, the birth of the eukaryotic virus realm can be envisaged as a turbulent process of mixing and matching of genes from several different sources, the most important one being, probably, the gene pool of prokaryotic viruses, in the melting pot of the emergent eukaryotic cell. At this juncture, we must, once again, stress the importance of the links between viral and cellular evolution and the coherence of the emerging scenarios. Indeed, the cataclysmic events of eukaryogenesis appear to have precipitated the birth of a new domain of the virus world. Of course, our models of the virus world evolution depend on the adopted scenario for evolution of cells; to wit, a "eukaryotes first" scenario [64,65] would call for a very different perspective on viral evolution as well. As explicated above, comparative-genomic data do not seem to be compatible with this scheme at all but, instead, strongly favor an archaeo-bacterial fusion scenario which dictates the polarity of the virus world evolution discussed in this section. Moreover, the apparent mixed contribution of bacteriophages and archaeal selfish elements to the formation of the genomes of some eukaryotic viruses, such as the NCLDV (Table 5), provides supportive feedback for the fusion models of eukaryogenesis.

## Conclusion

### The ancient Virus World and its connections to the evolution of cells

Our principal conclusions are encapsulated in the concept of the ancient Virus World. We envisage this world as a distinct flow of genes that emerged from within the primordial gene pool and retained its identity ever since, the numerous gene transfers between viral and cellular genomes notwithstanding. The notion of the virus world, one of its central tenets being intense gene mixing at the precellular stage of life's evolution, explains the spread of several essential genes (which we dubbed viral hallmarks) among apparently unrelated groups of viruses, observations that otherwise might seem completely baffling. The virus world concept blurs the distinction between monophyletic and polyphyletic origins of virus classes and implies a "third path". The major classes of viruses do not have a common origin in the traditional sense but neither are they unrelated, being connected through overlapping arrays of common genes, some of which are hallmarks of the Virus World.

The Virus World, apparently, remained continuous in time, in terms of uninterrupted inheritance of substantial sets of virus-specific genes, since its emergence from the primordial gene pool at the precellular stage of evolution to this day. The temporal continuity of the Virus World is complemented by its current interconnectedness as demonstrated by numerous observations of intervirus HGT, especially, the rampant gene exchange among phages [93-95]. The routes of HGT within the virus world can be most unexpected as spectacularly illustrated by the recent discovery of the homology of the surface glycoproteins of herpesviruses (large dsDNA viruses) and rhabdoviruses (negative-strand RNA viruses) [96,97]. In yet another dimension, the discovery of extensive movement of viruses between environments [2] attests to the spatial continuity of the virus community on earth. Hence the Virus World appears to be a spatial-temporal continuum that transcends the entire history of life on this planet.

The continuity of the Virus World notwithstanding, during eukaryogenesis, one of the most remarkable, turbulent transitions in life's evolution, it has gone through a second melting pot that gave rise to the realm of eukaryotic viruses. In this new domain of the Virus World, a few primeval lineages (primarily, viruses with small genomes) retained their identity, whereas most have been remolded into novel ones, albeit linked to the prokaryotic domain of the virus world through a variety of genes.

Despite the upheaval associated with eukaryogenesis, we submit that, in the Virus World, all viruses evolve from other viruses, even if via circuitous routes: *Omnis virus e virus*. Granted, this requires an important caveat: viruses do exist that seem to be disconnected form the rest of the virus world. Examples include several viruses of hyperthermophilic Crenarchaeota [11] and some of the insect polydnaviruses [98]. In terms of the classes of virus genes delineated here (see Table 2), the genomes of these viruses consist, mainly, of class 1 genes (relatively recent acquisitions from the host) and class 3 genes (ORFans). These viruses, although they are exceptions rather than the rule, might be viewed as a challenge to the Virus World concept, and their genome composition seems to lend some support to the escaped-genes hypothesis. However, given the strong evidence of the continuity of the virus world provided by the results of comparative-genomic analyses of other viruses, we are inclined to believe that these agents evolved from other, "normal" viruses via step-by-step gene substitution and loss of original viral genes. The escaped-genes scenario for the origin of these viruses could be falsified by delineation of the path leading from "normal" to "unique" viruses via gradual loss of hallmark genes in related viral genomes. Hints at such a solution might be coming from the discovery of the JRC and the hallmark packaging ATPase in some of the crenarchaeal viruses [11,54] and of the latter gene in one of the polydnavirus genomes (EVK, unpublished observations).

Having formulated the concept of the ancient Virus World, it makes sense to define the denizens of this world in more biologically relevant terms. The most meaningful definition appears to be that the Virus World consists of selfish genetic elements whose reproduction depends on certain basic capacities of cellular life forms, such as translation and energy transformation. This is an inclusive definition that accommodates not only traditional viruses but also a plethora of other selfish genetic elements, in particular, viroids that do not even have genes, and various kinds of mobile elements that integrate into the host genomes but propagate in the selfish mode. Once such elements lose their ability to reproduce autonomously, they emigrate from the virus world to the cellular world. Most often, having no function, they perish upon immigration but, on some occasions, the deteriorating selfish elements are functionally integrated into the host genome (especially, regulatory regions) which could be an important factor of the host's evolution [99,100]. Under this concept, the eukaryotic spliceosomal introns are a major incursion of the virus world into the cellular world. Indeed, the selfish nature of self-splicing group I and group II introns and their legitimate place in the virus world are evident, and the myriad spliceosomal introns of eukaryotes are believed to have evolved from group II introns, probably, coming from the mitochondrial endosymbiont, at the dawn of eukaryotic evolution [70,101,102]. In complex eukaryotes, such as mammals, introns comprise ~30% of the genome; another ~60% of the genome consists of intergenic regions that are chock-full of mobile elements in various stages of decay. Thus, paradoxically, the bulk of our genome seems to be derived from the ancient Virus World.

A crucial aspect of the Virus World concept is its inextricable connection with certain classes of models for the evolution of cells. In particular, the results of viral comparative genomics appear to demand a complex, pre-cellular stage of life's evolution, characterized by extensive mixing and matching of genetic elements, and an archaeo-bacterial fusion model of eukaryogenesis. Under this view, the various replication and expression strategies employed by viruses (Fig. 1) emerge as relics of an ancient, "experimental" stage of evolution. Two distinct versions of one of these experimental designs, the invention of dsDNA replication, were particularly successful – so much so that they gave rise to the two cell types that survived throughout the rest of life's history. It is interesting to note that this concept of pre-cellular evolution reverberates with Haldane's early vision: "...life may have remained in the virus stage for many millions of years before a suitable assemblage of elementary units was brought together in the first cell" [47]. The concept of the virus world and its connections with the evolution of cells has an important corollary: further mapping of the viral world will not only reveal the vagaries of virus evolution but should substantially inform the study of the origin of different types of cells.

## Reviewers' comments

### Reviewer's report 1

W. Ford Doolittle, Dalhousie University

Fond as I am of many of Dr. Koonin's imaginative ideas, I'm not so keen on this one. My reasons are three-fold.

First, Koonin *et al*. acknowledge that HGT among and between viruses and hosts is one of the major forces in the evolution of their genomes. Their viral hallmark genes have had plenty of opportunity in the last three-four billion years to make their way into one or another host genome. That they have not done so must mean that the proteins they encode are of no use to hosts. And if they are of no use to hosts, then that in itself is sufficient explanation for their exclusive presence in viruses. It is irrelevant to the question of how long these genes have been associated with viruses.

**Author response: ***Certainly, this is an ingenious and pertinent argument. However, matters are not quite so simple. Firstly, it is not absolutely true that cellular life forms have not recruited viral hallmark genes for their own purposes. A clear counterexample (it is discussed in the present paper but I will rehash the issue in brief) is the telomerase, a reverse transcriptase that apparently has been acquired by an ancestral eukaryote from a mobile retroelement and employed in an essential eukaryotic function, the replication of chromosomal ends. Let us note the pattern here: reverse transcriptase is a hallmark of the virus world that is common in both prokaryotic and eukaryotic selfish elements but performs an essential cellular function in eukaryotes only. It is hard to deny that this pattern is strongly suggestive of the origin of the gene in question within the virus world, with subsequent recruitment for the cellular function (telomerase) in eukaryotes. Granted, this is an exception. Most of the viral hallmark genes, indeed, are amiss from cellular life forms, which does seem to suggests that they are of no use to cells or might even be deleterious. In some cases, e.g., the JRC, this is understandable (cells do not need capsids), in others, the reasons remain mysterious, e.g., S3H. However, is exclusive presence of these genes in a broad variety of viruses (and this is how the hallmark genes are defined), indeed, irrelevant for the antiquity of their association with viruses? Certainly, the hallmark genes must have entered the virus world by one route or another, whenever that might have happened, time-wise. If the source of these genes was not the primordial gene pool, they must have come from genomes of cellular organisms. That means that these genes, at some time, have been of some use to cellular organisms. It seems extremely far fetched to suppose that, after being acquired by a virus, these genes all of a sudden became useless for cells. A different, probably, more viable version of a late, cellular origin of the hallmark genes might be considered (as, indeed, pointed out by Doolittle in the second part of his comment). This alternative involves acquisition of the progenitor of a hallmark gene from a cellular genome by a particular virus accompanied by a dramatic acceleration of evolution caused by the functional alteration. The gene, then, would sweep the viral world. The assumption here is that there is a set of functional constraints that is common to a variety of viruses. This seems to be plausible for some of the hallmark genes but not others, e.g., it does stand to reason that the capsids have common design even in very different viruses but, in the cases of S3H or the primase, a viral common denominator hardly can be gleaned, which does not bode well for the HGT scenario. We continue with this line of reasoning in our response to Doolittle's second point*.

Second, and again, Koonin *et al*. acknowledge the role of HGT between viral "lineages" in the evolution of their genomes. The presence of genes in diverse viruses, though consistent with their presence in some LUCAV or primordial gene pool, is not proof of it. These genes could have arisen in one lineage and been transferred to others. By "arisen" I mean evolved so dramatically away from whatever viral or host function they used to perform that we can no longer detect the relationship. This headlong erasing-all-traces-of-the-past phase would have eased up before the born-again genes began their inter-viral odyssey, explaining the "conservation of the hallmark proteins in extremely diverse classes of viruses with widely different replication/expression strategies." Koonin *et al*. dismiss the HGT scenario because of "the extremely distant similarity between the hallmark proteins from diverse virus groups". But they are not so extremely distant that their homology is undetectable by sequence, and no one says the HGTs have to have been recent. Koonin would not, I think, argue that the extreme dissimilarity (homology not detectable by sequence) of many eukaryotic proteins to any in prokaryotes argues for their primordial pre-cellular origin.

**Author response: ***This point is very closely related to the first one and their separation might be somewhat arbitrary. Nevertheless, we follow the structure of Doolittle's argument and respond separately. First of all, let us notice that, beyond any doubt, HGT is an extremely important aspect of viral evolution. Not only do not we deny the role of HGT but, on the contrary, we emphasize that HGT is the glue that holds the virus world together. Furthermore, it is likely that the currently observed distribution of the hallmark genes among viruses has been affected by HGT. However, we still maintain that HGT is not the preferred and, indeed, not a good explanation at all for the general pattern of that distribution. We attempted to present the arguments in this article but let us consolidate them here. 1. The distant similarity between the versions of the hallmark genes in diverse viral lineages suggests the ancient spread of these genes in the virus world. However, we must agree with Doolittle that ancient here does not necessarily equal primordial. 2. The essentiality of the functions of the hallmark genes is not well compatible with HGT being the principal mode of their dissemination over the virus world. Indeed, a question seems inevitable: HGT of a hallmark to what? Without the given hallmark gene, e.g., the JRC or the RdRp, there would be no competent recipient virus. Thus, the HGT scenario necessarily would involve displacement of ancestral genes with the same function, e. g., of a rod-shaped capsid protein gene by the JRC gene or of a gene for a DNA polymerase with the RCRE gene. Such displacements are not impossible in principle but some will turn out to be awkward when the pre-existing viral replication machinery is poorly suited to accommodate the newcomer. More importantly, what kind of virus world does this translate into? Seemingly, one with perpetual displacement of essential genes as a result of rampant HGT. Where would these essential genes come from in the first place? They would be there as long as viruses exist – suspiciously similar to the model of the virus world discussed in this paper. 3. The conservation of the hallmark genes between prokaryotic and eukaryotic viruses seems to be another telltale sign of primordial origin of these genes. We are unaware of any extensive gene movements between prokaryotic and eukaryotic viruses in modern times. However, at some point(s) in the past, such exchanges must have happened, and as discussed in this paper, the epoch of eukaryogenesis seems to be the most likely period for these events to occur (the second "melting pot" of virus evolution). Should that be the case, the spread of hallmark genes across the virus world is at least as old as the eukaryotes. However, that is a completely unrealistic upper bound because the hallmark genes must have been already contained in bacteriophage genomes in order to contribute to the emergence of the eukaryotic viruses. Taking the reasonable uniformitarian approach to bacterial evolution, we are justified to deduce that the prevalence of the hallmark genes in the virus world is as old as bacteria. From there, it is but a small – though not necessarily easy, given that uniformitarianism is hardly applicable here, – step back to the primordial gene pool*.

*The final point on this second comment of Doolittle is about eukaryotes and whether or not we would take the existence of eukaryotic proteins without detectable prokaryotic homologs (or with extremely distant homologs) as evidence of a primordial, pre-cellular emergence of eukaryotes. Surely, we won't although it is notable that others make this argument quite earnestly (perhaps, not exactly pre-cellular origin of eukaryotes but, definitely, a distinct, primordial eukaryotic lineage – see *[60]*and references therein). The difference from the situation with viruses is clear and straightforward: it is demonstrable in many cases and seems highly plausible in others that emergence of novel eukaryotic functions entails major acceleration of the evolution of the genes that were inherited from prokaryotes but were exapted for these novel functions (the cytoskeleton and the ubiquitin system are obvious cases in point). Once emerged, the novel, eukaryote-specific cellular structures were rapidly fixed and then changed minimally throughout the evolution of eukaryotes. Thus the acceleration of evolution was dramatic but very brief, explaining the chasm between highly conserved, pan-eukaryotic proteins and their (sometimes, barely recognizable) prokaryotic progenitors. In our view, this is, by far, a simpler, better explanation of the observed pattern than any claim of a "new entity", let it be cellular or pre-cellular. The case of viruses is in a stark contrast: no pan-viral genes, no perceptible set of common functional constraints across diverse viral lineages (with some possible exceptions like JRC), hence no basis for rapid acceleration upon the entrance of a hallmark gene into the viral world followed by fixation in the new functional niche and the accompanying, equally dramatic deceleration of evolution. In a sense, this is the crux of our argument: viral hallmark genes are altogether a different lot from the sets of conserved pan-eukaryotic genes (or pan-archaeal, or pan-bacterial ones). Hence a qualitatively different evolutionary scenario is called for, and we try to step up to the plate in this paper*.

Third, I totally agree with Koonin *et al*. that the habitual dismissal of an early viral origin "on the grounds that all extant viruses are intracellular parasites" is jejune by any standards, and that virus-like entities surely predated the appearance of modern cells. But today's viruses do not have to descend directly – in the sense that any of their genes descend directly – from these entities. Adam's sins are not my sins, even though I'm pretty sure the lineage of sinning is unbroken.

**Author response: ***It is important that we agree on the antiquity of virus-like entities and (presumably) their importance in the evolution of life from the get go. We also do not disagree on the possibility that today's viruses have nothing to do with those of old. What we do disagree about is the conception that this possibility is as realistic as the alternative outlined in this paper, namely, that the major lineages of modern viruses derive directly from the primordial, pre-cellular gene pool. Not only is there no shred of evidence in support of the presumed sweep of new genes over the virus world but there are (we believe) substantial arguments against specific scenarios that must be developed to make such a sweep credible. These arguments are summarized in the paper and in our responses to Doolittle's first two points. Thus, we believe that the origin of the viral hallmark genes and several major lineages of viruses directly from the primordial gene pool is the simplest explanation of the patterns discovered by comparative genomics of viruses, and this scenario shows strong synergy with specific models of cell evolution. The epistemological status of this conclusion is briefly considered below*.

All that said, I don't disfavor publication of this ms. Evolutionary scenarios are an artform. They usefully exercise the brain, causing us to look at old data in new ways and stimulating us to collect new data. They do not have to be true!

**Author response: ***It might be wise to refrain from an explicit philosophical discussion and simply take this last statement of Doolittle as a legitimate opinion which it is. However, we strongly (inasmuch as the very notion of a "strong disagreement" is still relevant in post-postmodern philosophy) disagree with this agnostic stance (which we take as being serious rather than ironic) and think that this outlook does not help studies of early evolution. To be more explicit, we do not accept that "evolutionary scenarios are an artform" but rather contend that this is a distinct and important area of research within the general domain of historical sciences, such as evolutionary biology and cosmology. These scenarios do not have to be true, i. e., they do not have to be and never can be precise, proven accounts of the events that actually happened, but have to be earnest and defendable attempts on attaining an approximation of the truth that is, at least in some aspects, closer than previously available approximations. That is, we believe, the justification of research into such scenarios rather than the benefits of intellectual workout that accompanies these efforts*.

*Presumably, the notion that evolutionary scenarios are not a form of science stems, primarily, from the apparent lack of Popperian falsifiability for these concepts. There is, however, a lot to say about the status of such scenarios vis-à-vis the Popperian model of science and about the validity of that model and its applicability, especially, in the domain of historical sciences. First of all, the notion that evolutionary scenarios are unfalsifiable needs to be clarified. There are specific, falsifiable predictions in any evolutionary scenario worth its salt. To use an obvious example from the present work*, Omnis virus e virus *is an important part of our general concept of viral evolution, and it can be falsified by the discovery of a clear case of the origin of a virus from escaped genes. It is true that the scenarios are not falsifiable in their entirety, and neither is any historical narrative (the same applies to many generalizations of non-historical sciences – indeed, it is quite dubious that a general Popperian model of science is realistic – see, e.g., Godfrey-Smith, Theory and Reality: An Introduction to the Philosophy of Science). We believe that, in general, the verificationist framework is more relevant as the epistemological foundation of the research into fundamental aspects of early evolution. More specifically, we think that the "complete evidence" approach (more or less, sensu Carnap), i.e., convergence (consilience) of various lines of evidence, none of which might be compelling in itself, has the potential of rendering some scenarios of early evolution substantially more likely than others – on some occasions, to such an extent that they closely approach the status of "truth". Again, these scenarios should and do include specific falsifiable hypotheses but the validity of the construct as a whole can only be established in terms of likelihood and only by synthesis of a multitude of evidence. An obvious example is the "RNA World" – an extremely bold generalization on early stages of life's evolution but one that is, by now, more or less universally accepted, on the strength of converging evidence on the activities of RNA in modern life forms, ribozyme chemistry, and the logic of evolution. In this paper, we tried to show the convergence of widely different lines of evidence that make the concept of the ancient virus world a plausible one*.

### Reviewer's report 2

J. Peter Gogarten, University of Connecticut

The manuscript by Koonin *et a*. describes a scenario for virus evolution that links the origin of virus to the early evolution and origin of cells. In particular, the authors suggest that viruses and phages are descendents of selfish genetic elements that were already present before the evolution of cells and genomes. The argument is based on the wide, but not universal, distribution of viral "signature" genes, and agrees with hypercycle models of early molecular evolution that showed that these early networks are prone to infection by molecular parasites. The basic hypothesis presented in this manuscript is reasonable, well developed and provides a good alternative to the scenarios that describe virus' origins as genes escaped from cellular organisms.

The manuscript treats the early evolution of viruses as a speculative topic. Given that evolution is traced back to the origin of cells, this might appear justified; however, I find the article more speculative than necessary. The authors link virus origins to one particular model of cellular origins and early evolution, the authors chose the scenarios by Martin, Muller, and Russell (see my references 1 & 2). This leads to unnecessarily detailed speculation. These models made an important contribution in detailing possible pathways to cellular life and towards the eukaryotic cell, but many alternative syntrophic relationships at the root of the eukaryotes were suggested, for examples see my refs. 3–6. Furthermore, some details of the scenario followed in this manuscript have been debated in the past (e.g., molecular phylogenies do not indicate a close relationship between eukaryotes and methanogenes); an RNA based genome might not necessarily be less complex, because early RNA polymerases were inferred to be error correcting (see my ref. 7); and the assumptions that the authors make for the most recent common ancestor of bacteria, archaea and eukaryotes contradict much of what was learned about early evolution during the previous decades: Molecular evidence points towards energy coupling membranes being already present in the MRCA of all known life (see discussion in my ref. 8). Apparently, the MRCA of all known cellular organisms was not devoid of membranes, but already had a complex targeting machinery for membrane proteins (my ref. 9), terminal oxidases (my ref. 10 and Simonetta Gribaldo, pers. communication) and ATP synthases driven by transmembrane electricochemical ion gradients (my ref. 11). All of these systems apparently predated the MRCA of all known cellular organism. The idea of a primitive, pre-cellular common ancestor dates back to the first molecular trees of life, when Fox and Woese concluded that this organism might have been a progenote, i.e. an organism without a tight coupling between geno- and phenotype (my refs. 12 & 13). While a pre-cellular organism with a distributed, communal genome likely was a stage in early cellular evolution, molecular evidence suggests that at the time of the organismal MRCA cellular structures were much more advances than envisioned in the scenario described by the authors. Horizontal gene transfer complicates a simple back extrapolation, but the molecular phylogenies of ATP synthases, elongation factors, ribosomes and signal recognition proteins are in surprising agreement, suggesting that with few recognizable exceptions, the genes in question were transferred only between closely related organisms. One way to arrive at a more primitive MRCA of the three domains is to place the root of the tree of life on the eukaryotic branch as suggested by Forterre and collaborators (my ref. 14). However, several shared derived characters of ATPases (my ref. 15) and elongation factors (my ref. 16) suggest that the root is located outside the clade comprised by the archaea and the eukaryotic nucleocytoplasm.

A frequent argument in favor of a pre-cellular MRCA, also invoked in the present manuscript, is based on the different lipid and cell wall composition of archaea, bacteria and eukaryotes. I think this is a red herring: All three domains synthesize isoprenoids (and while some of the enzymes are different between the two domains, the pathway as a whole and some of the enzymes appear to be homologous) (my refs. 17 & 18); furthermore, all cells use polyprenols like dolichol to transport sugars through membranes (either as activated cell wall precursors or for glycosilation reactions inside the ER or in the periplasmic space), indicating that the ability to synthesize long chain branched aliphatic alcohols was around early; and S-layer proteins are considered by some as the likely ancestral cell wall material (my ref. 19). If the ester linked fatty acid based membrane lipids were a later bacterial invention, it would not be surprising to find this pathway also in eukaryotes, because all known eukaryotes apparently evolved from ancestors that once possessed mitochondria (my ref. 20).

While I do not agree with some of the details of the described scenario for cellular evolution, these details are not crucial to the central thesis of the manuscript (there is no arrow in figure 2 that connects the MRCA of all life and the bacterial and archaeal MRCAs to the virus world). The proposed hypothesis on the origin of viruses depends only on the presence of a progenote stage in early evolution, regardless whether this stage was part of the "stem" leading to the organismal MRCA, or whether this stage was reached independently by the lineages leading to the archaeal and bacterial domains.

**Author response:***We agree with Gogarten that our concept of the ancient virus world does not critically depend on the nature of LUCA (MRCA of all modern cells); what is actually required is an advanced, diverse pre-cellular pool of genetic elements. Indeed, this is a useful point to make and we do so explicitly in the revised manuscript. It might be useful to note that the pre-cellular stage of evolution in our model does not seem to be the progenote (Woese, Fox, 1977, J. Mol. Evol. 10: 1–6) in the original sense because that latter was supposed to possess a primitive, imprecise translation system which would not work for the level of pre-cellular complexity envisaged here*.

*Since the nature of LUCA is not central here, it is not the place to present the argument for a pre-cellular LUCA that has been discussed previously *[37]*and, more briefly, in this paper. Just in a nutshell: we do not believe that the use of non-homologous pathways for lipid biosynthesis by archaea and bacteria is a "red herring"; on the contrary, it is a major conundrum in need of a solution. Yes, all bacteria do synthesize isoprenoids, and some of them do so with the use of the archaeal enzymatic machinery, probably, acquired via HGT. Whether or not the classical bacterial pathway of isoprenoid biosynthesis derives from a common ancestor with the archaeal pathway is a more complex matter. What is important, however, is that bacteria never use isoprenoids to build their membranes. So the notion of a cellular LUCA would require displacement of the ancestral, archaeal-type, isoprenoid-based membrane by the newly emerged, bacterial-type fatty-acid-based membrane, without elimination of the isoprenoid biosynthesis pathway that would then serve other functions (as they do in modern bacteria). Not an impossible scenario in itself but a mechanistically challenging one, and with the underlying selective forces utterly mysterious*.

**Gogarten responds in a second review: **I fail to see a mechanistic challenge. To a large extent lipids based on fatty acids, long chain alcohols, and even non-biogenic lipids, for example extracted from the Murchison meteorite (my ref. 21), appear mechanistically equivalent.

**Author response:***However, the lipid argument is not the only one for a non-cellular LUCA. The lack of homology between the core components of the DNA replication machineries in archaea and bacteria, which implies a fragmented RNA genome in LUCA, is equally important. This effectively rules out accurate genome segregation and does not bode well for a cellular LUCA at all. We certainly do not claim to "know" what LUCA was like but we do perceive the non-cellular model discussed in *[37]*to be the current solution of choice*.

**Gogarten responds in a second review: **Even an RNA based genome might have been less fragmented than assumed (see above), furthermore the lack of perceived sequence homology between the bacterial and archaeal/eucaryal DNA replication machinery could be due to divergence, not lack of shared ancestry. Functionally the processes and sub-processes in the replication fork are very similar in all three domains of life, which seems to be difficult to explain by convergent evolution.

Alternative explanations for the features that were used to argue for a non-cellular MRCA exist; in contrast, the findings that indicate a cellular MRCA of the three domains (e.g., the machineries used in chemiosmotic coupling, and for the targeting of membrane proteins apparently predate the MRCA of the three domains, see above) at present have not been reconciled with a non-cellular entity. Therefore, at present a pre-cellular MRCA of the three domains (LUCA) appears at odds with the available data. I do not perceive this scenario as the solution of choice.

**Gogarten first review continues**: Other suggestion:

Add additional citations: To me the idea that virus and phage evolution began early in the evolution of life appears very reasonable, and I would be surprised if others had not formulated similar ideas in the past.

*Author response: We believe that the notion of the virus world as explicated here is new. The idea of a primordial origin of virus-like entities, of course, is old, even if unpopular lately (at least prior to the work of the Bamford group on the JRC structure in diverse viruses and the discovery of the mimivirus – all this is cited here). We cite the classic textbook of Luria and Darnell *[40]*which offers an insightful discussion of the early ideas in this area. In the revision, we added the citation of Felix D'Herelle's 1922 book which is where the idea that viruses might precede cells in evolution, probably, was proposed for the first time *[46].

**Gogarten responds in a second review: **The addition of the *D'Herelle *citation is an excellent choice, the following might be interesting as well, it seems more similar to the ideas developed in the manuscript: According to Sapp (my ref. 22) the idea of early co-existence of viruses and cells was expressed by Peter Raven in a letter to R. E. Buchanan on November 3, 1970 "*Raven suggested that viruses, probably as old as life itself, might be regarded as by-products of bacterial reproduction, in which segments of DNA or RNA protected with protein coats spread from cell to cell, directing the host cell's metabolism to reproduce more of the viral DNA or RNA*."

**Gogarten's first review continues**: Section on "*The primordial gene pool: the crucible of the major virus lineages****", last paragraph: ***Why would the transfer need to be rampant? The connection to a **non**-cellular model for early evolution could be better developed. Pre-cells, or cells with small, possibly partial genomes (my ref. 23) should do just fine for the indicated stages, as long as there is a moderate level of transfer allowing for recombination and for molecular parasites to evolve.

**Author response***: We softened this statement in the revision. Still, to account for the observed spread of the hallmark genes, gene trafficking between different types of genetic elements must have been much more intense than anything observed in modern life forms, and we suspect that a moderate level of transfer between cellular entities won't do*.

### Reviewer's report 2: reference list

1. Martin W, Russell MJ: **On the origins of cells: a hypothesis for the evolutionary transitions from abiotic geochemistry to chemoautotrophic prokaryotes, and from prokaryotes to nucleated cells**. *Philos Trans R Soc Lond B Biol Sci *2003, **358**(1429):59–83; discussion 83-55.

2. Martin W, Muller M: **The hydrogen hypothesis for the first eukaryote**. *Nature *1998, **392**(6671):37–41.

3. Searcy DG: **Origins of mitochondria and chloroplasts from sulfur based symbiosis. **In: *The Origin and Evolution of the Cell*. Edited by Hartman H, Matsuno, K.: World Scientific; 1992: 47–78.

4. Margulis L: **Symbiosis in Cell Evolution: Microbial Communities in the Archean and Proterozoic Eons**, 2nd edn: W H Freeman & Co; 1995.

5. Searcy DG: **Metabolic integration during the evolutionary origin of mitochondria**. *Cell Res *2003, **13**(4):229–238.

6. Lopez-Garcia P, Moreira D: **Metabolic symbiosis at the origin of eukaryotes**. *Trends Biochem Sci *1999, **24**(3):88–93.

7. Poole AM, Logan DT: **Modern mRNA proofreading and repair: clues that the last universal common ancestor possessed an RNA genome? ***Mol Biol Evol *2005, **22**(6):1444–1455.

8. Gogarten JP, Taiz L: **Evolution of proton pumping ATPases: Rooting the tree of life**. *Photosynthesis Research *1992, **33**:137–146.

9. Gribaldo S, Cammarano P: **The root of the universal tree of life inferred from anciently duplicated genes encoding components of the protein-targeting machinery**. *Journal Of Molecular Evolution *1998, **47**(5):508–516.

10. Castresana J, Lubben M, Saraste M, Higgins DG: **Evolution of cytochrome oxidase, an enzyme older than atmospheric oxygen**. *Embo J *1994, **13**(11):2516–2525.

11. Gogarten JP, Kibak H, Dittrich P, Taiz L, Bowman EJ, Bowman BJ, Manolson MF, Poole RJ, Date T, Oshima T *et al*: **Evolution of the vacuolar H+-ATPase: implications for the origin of eukaryotes**. *Proc Natl Acad Sci USA *1989, **86**(17):6661–6665.

12. Woese CR, Fox GE: **Phylogenetic structure of the prokaryotic domain: the primary kingdoms**. *Proc Natl Acad Sci USA *1977, **74**(11):5088–5090.

13. Woese CR, Fox GE: **The concept of cellular evolution**. *J Mol Evol *1977, **10**(1):1–6.

14. Forterre P, Philippe H: **Where is the root of the universal tree of life? ***BioEssays *1999, **21**(10):871–879.

15. Zhaxybayeva O, Lapierre P, Gogarten JP: **Ancient gene duplications and the root(s) of the tree of life**. *Protoplasma *2005, **227**(1):53–64.

16. Skophammer RG, Herbold CW, Rivera MC, Servin JA, Lake JA: **Evidence that the Root of the Tree of Life Is Not within the Archaea**. *Mol Biol Evol *2006, **23**(9):1648–1651.

17. Boucher Y, Kamekura M, Doolittle WF: **Origins and evolution of isoprenoid lipid biosynthesis in archaea**. *Mol Microbiol *2004, **52**(2):515–527.

18. Boucher Y, Doolittle WF: **The role of lateral gene transfer in the evolution of isoprenoid biosynthesis pathways**. *Mol Microbiol *2000, **37**(4):703–716.

19. Claus H, Akca E, Debaerdemaeker T, Evrard C, Declercq JP, Harris JR, Schlott B, Konig H: **Molecular organization of selected prokaryotic S-layer proteins**. *Can J Microbiol *2005, **51**(9):731–743.

20. Keeling PJ, Burger G, Durnford DG, Lang BF, Lee RW, Pearlman RE, Roger AJ, Gray MW: **The tree of eukaryotes**. *Trends Ecol Evol *2005, **20**(12):670–676.

21. Deamer DW: **Role of amphiphilic compounds in the evolution of membrane structure on the early earth**. *Orig Life Evol Biosph *1986, **17**(1):3–25.

22. Sapp J: **The prokaryote-eukaryote dichotomy: meanings and mythology**. *Microbiol Mol Biol Rev *2005, **69**(2):292–305.

23. Lawrence JG:**Gene transfer and minimal genome size**. In: *Size Limits of Very Small Microorganisms*. Washington, D.C: National Research Council.; 1999: 32–38.

### Reviewer's report 3

Arcady Mushegian, Stowers Institute

Section on "*Viral hallmark genes: beacons of the ancient virus world*", 4^th ^paragraph:maybe tread more carefully on LBA artifacts:if taken literally, and if virus enzymes are long branches, they would attract each other, would they not? (same applies to the argument in the 6^th ^paragraph of the same section).

**Author response:***yes, this is a good catch, the artifact involved here is not, exactly, LBA; the wording was modified*.

Section on "*Viral hallmark genes: beacons of the ancient virus world*", 5^th ^paragraph: do we indeed have the evidence that all viral JRC's are monophyletic, to the exclusion of nucleoplasmin and PNGase? (on the same matter, Table 3: 'protein-protein interaction domains of certain enzymes' is ambiguous: the enzymes in question have peptide substrates, so one should perhaps leave open the possibility of theancient relationship to a peptide-modifying enzyme – or disprove it more convincingly).

**Author response: ***The statement in question was softened. Obtaining such evidence for JRC is, indeed, extremely hard. Note, however, that nucleoplasmin and PNGase are exclusively eukaryotic proteins, in a marked contrast to the ubiquitous JRC. This seems to define the vector of evolution quite clearly. This is a subject for another day, though*.

Table 4: consider replacing "smacks of essentialism and might not be fruitful" by something like "does little to explain the trajectories of most other virus-specific and virus hallmark genes".

**Author response:***Appreciated; a hybrid version was substituted for the old text*.

General discussion item: Archaeal genomes themselves appear to be partitioned into unique (ultimately also eukaryotic) informational genes and bacteria-like operational genes. If this isalso to be understood asevidence for an ancient gene exchange, has there been a concomitant exchange of virus-like elements?

**Author response:***The notion of the partitioning of archaeal genes into two classes with distinct evolutionary provenances seems to be somewhat misguided (this is, of course, very regrettable because it comes from a well-known and, in many ways, still relevant paper of which one of us is the first author: Koonin et al. Mol Microbiol. 1997 Aug;25(4):619–37). However, it is actually eukaryotes that have a "bipartite" gene set, with the informational genes coming, predominantly, from archaea and the operational genes, mostly, from bacteria. In a three-way comparison, it is impossible to decide whether the partitioning applies to archaeal or to eukaryotic genomes but the notion of the symbiotic origin of eukaryotes breaks the symmetry. This being said, there was, of course enormous amount of HGT between bacteria and archaea, and this involved virus-like elements as well. This is readily demonstrated by comparative genomics of viruses of mesophilic euryarchaea and various archaeal plasmids. However, viruses of hyperthermophilic crenarchaeota are very distinct and seem to be a unique, almost isolated domain of the virus world (see Prangishvili et al. Virus Res. 2006 Apr;117(1):52–67)*.

Finally, I would like the authors to address the following. The difference between the proposed scenario and Forterre hypothesis is inthe two main respects: i. Forterre says that ancient cellular life had RNA genome, while the new hypothesis says that RNA genome was replaced by mixed RNA/DNA genome (and perhaps then by DNA genome) pre-escape from inorganic compartments, and ii. Forterre says DNA genome was invented by viruses to protect itself from the host defense, while the new hypothesis says that DNA genome was invented by the primordial pre-escape genetic ensemble in the compartments, perhaps as a physical stabilization measure, and did not favor viral over non-viral genomes, if indeed there was any difference. Is this an accurate summary? If so, perhaps the authors should emphasize not only the difference between theForterre and their own theories, though of coursesuch difference issignificant, but also similar points that set these two theories apart from all the previous ones – i.e., for example, similar views on early and polyphyletic origin of viruses, intertwined evolutionary history of viral and cellular LUCAs, etc.?

**Author response:***The summary of differences is pretty accurate; we might add the two (in our scenario) versus three (in Forterre's scenario) primary cellular lineages as another important distinction. In any case, the point is well taken, we agree that it is useful to emphasize some similarities to Forterre's views at the level of the most general meta-concepts, so language to that effect has been added in the revised text and *Table 4.

## Authors' contributions

EVK formulated the original hypothesis, collected and analyzed the data, and wrote the original draft of the manuscript; TGS and VVD made essential contributions to the hypothesis in its final form and participated in the subsequent revisions of the manuscript.
